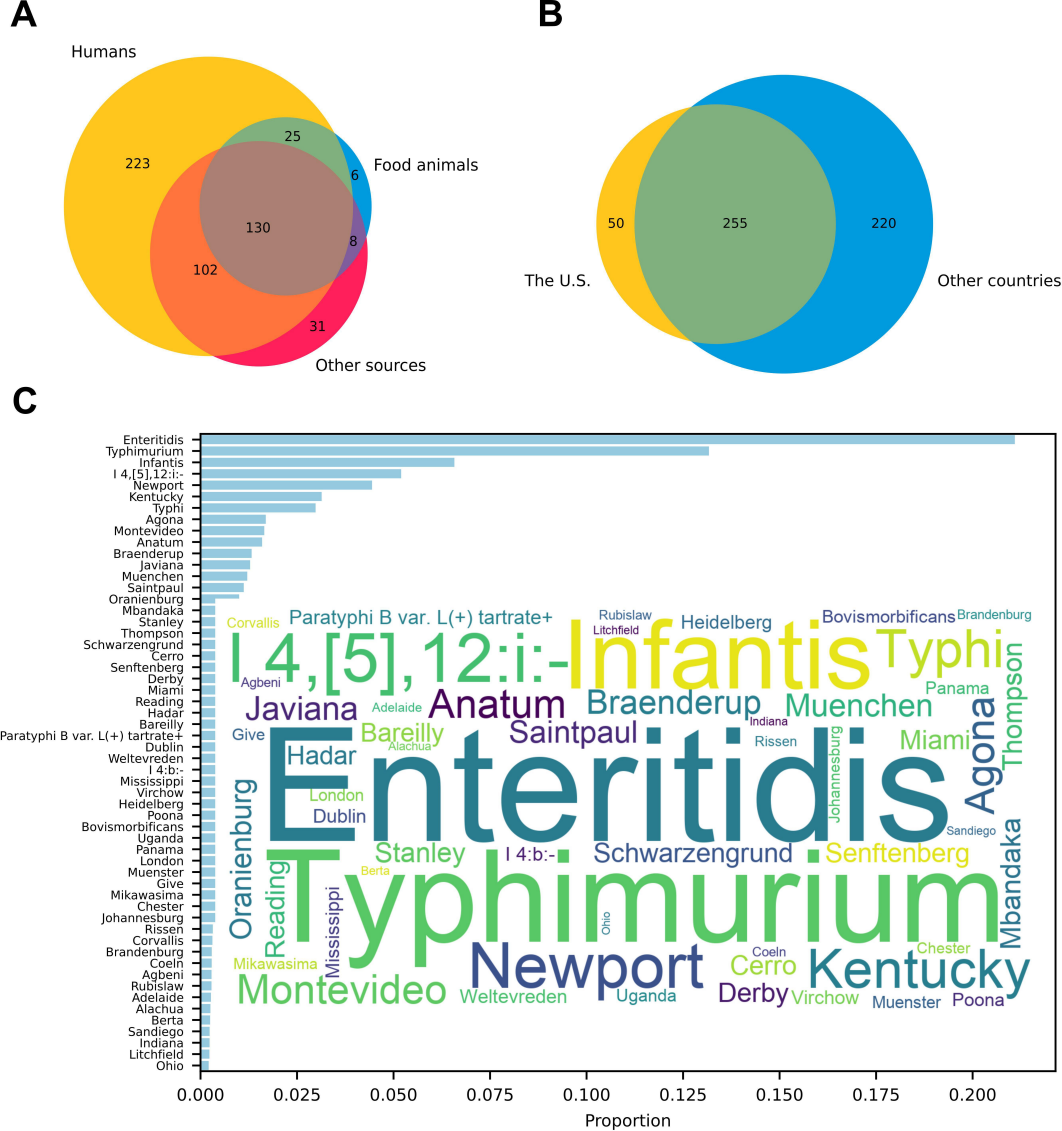# Articles of Significant Interest in This Issue

**DOI:** 10.1128/aem.00543-25

**Published:** 2025-03-19

## AN ASTROBIOLOGICAL PERSPECTIVE ON MICROBIAL BIOFILMS

In this Planetary Microbiology minireview, Gonzalez-Henao and Schrenk (e01778-24) discuss how biofilm markers could be used as biosignatures to assess the habitability of other planets.


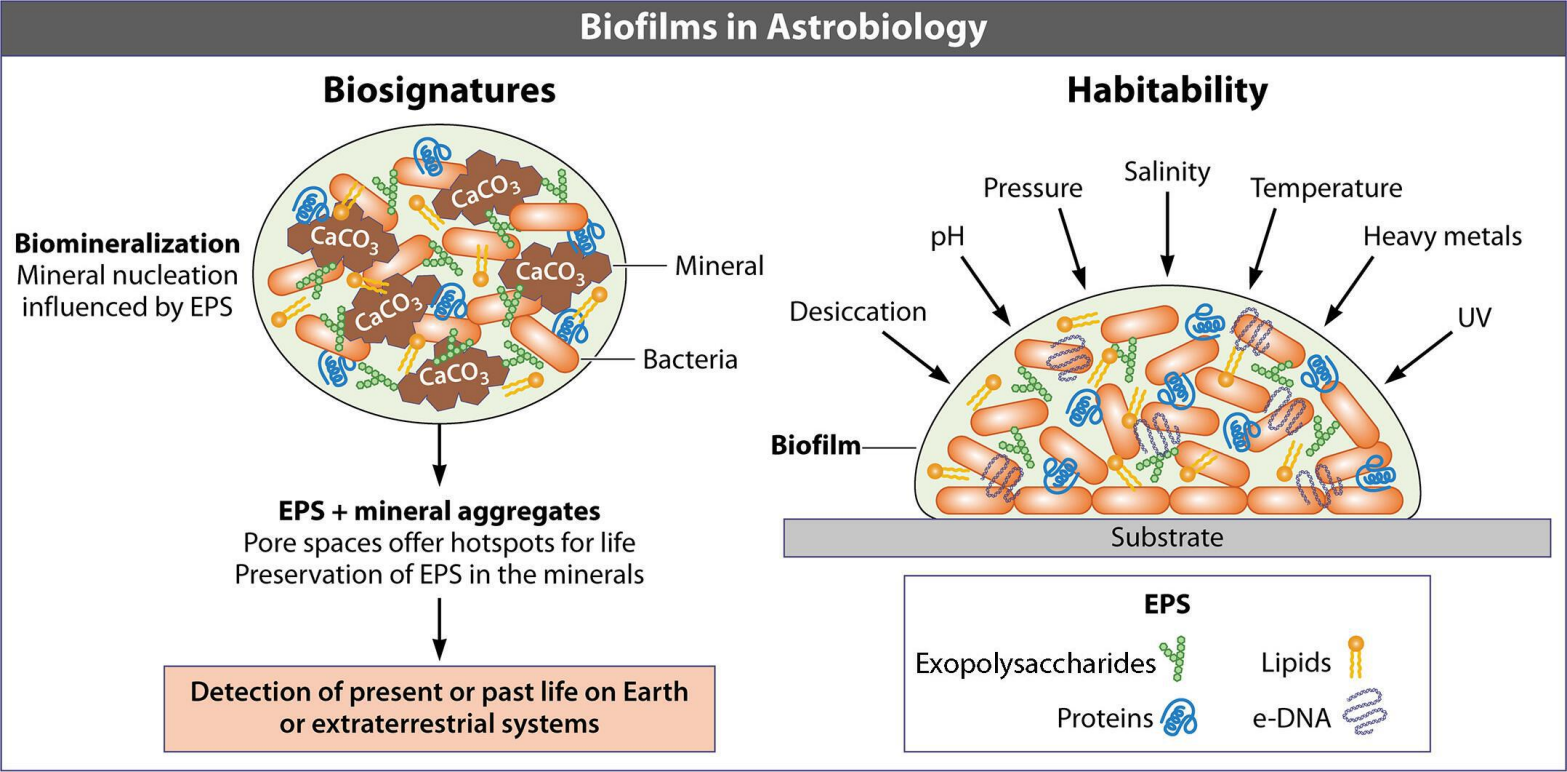


## THE DARK SIDE OF *TRICHODERMA* BIOCONTROL

Soil *Trichoderma* species used in crop health management can have pathogenic potential, as demonstrated by Pfordt et al. (e01931-24). This study underscores the need for biosecurity assessment and monitoring of *Trichoderma* strains in sustainable agriculture.


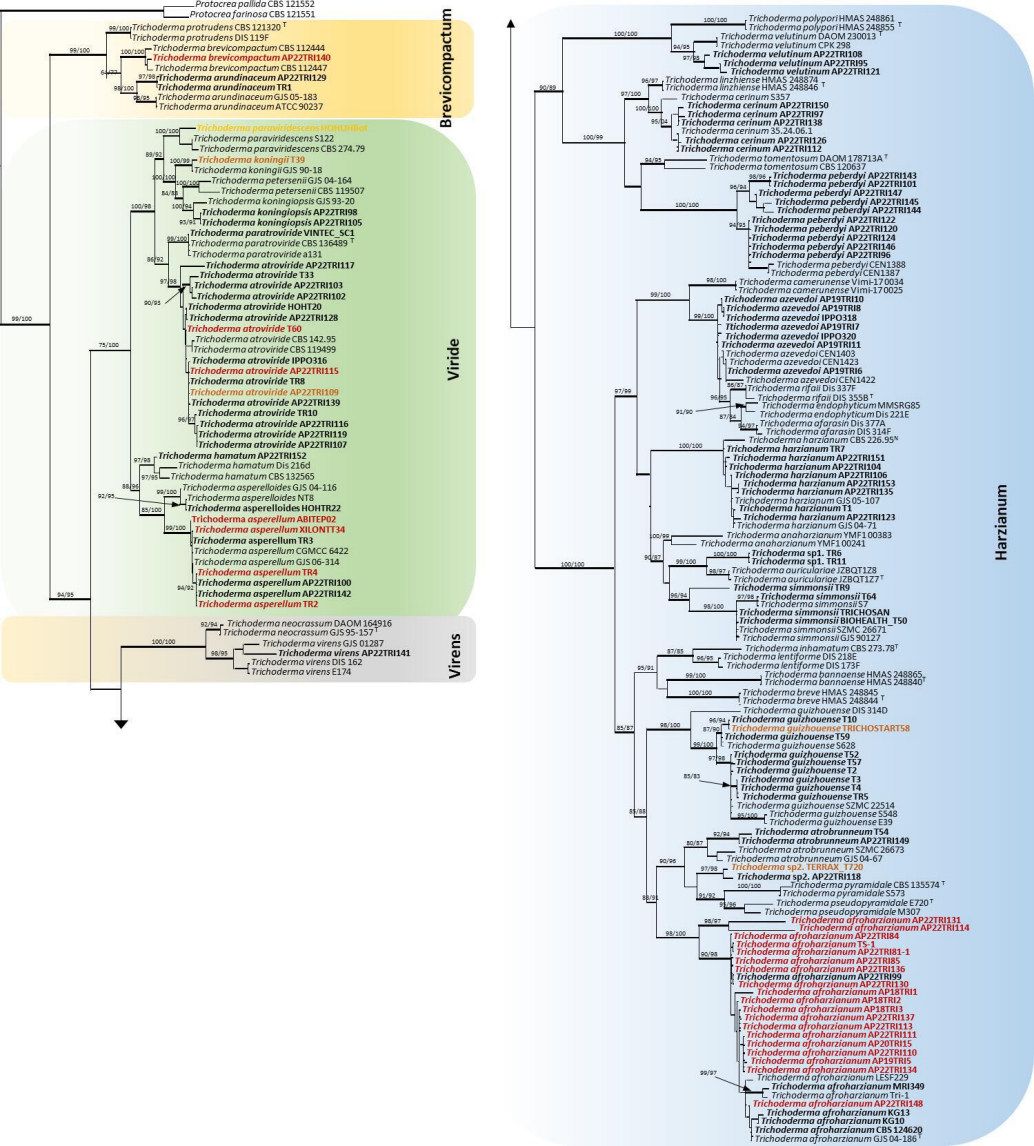


## RECOVERY OF SOIL MICROBES, ONE DROP AT A TIME

Dai et al. (e01794-24) describe a microfluidic method for the enrichment and recovery in pure culture of previously uncultured diversity of soil microorganisms.


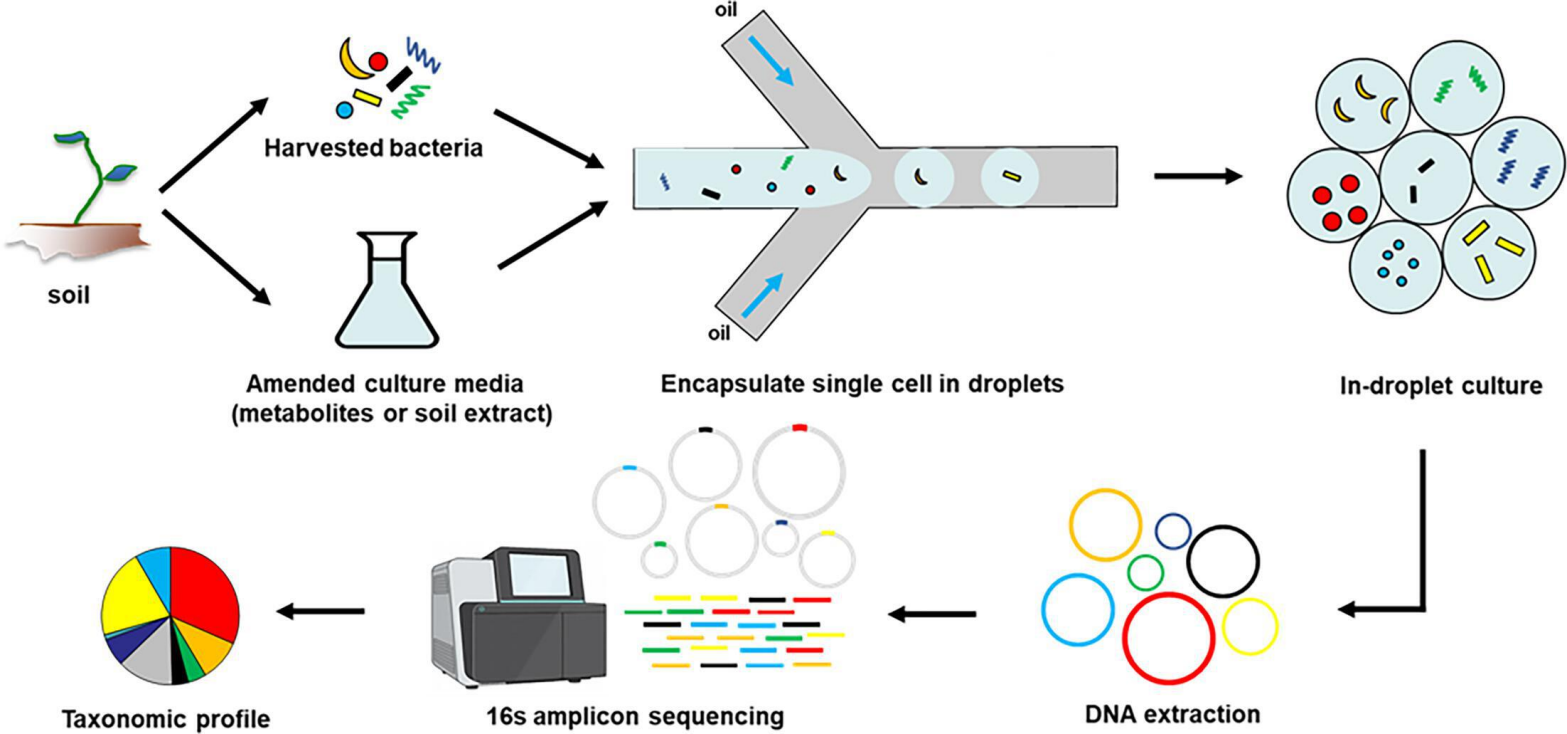


## COMPLETING THE “INCOMPLETE” TCA CYCLE OF *THERMOCOCCUS KODAKARENSIS*

Growth experiments and tracer-based metabolomics are used by Su et al. (e02017-24) to elucidate how *Thermococcus kodakarensis* survives with an “incomplete” TCA cycle.


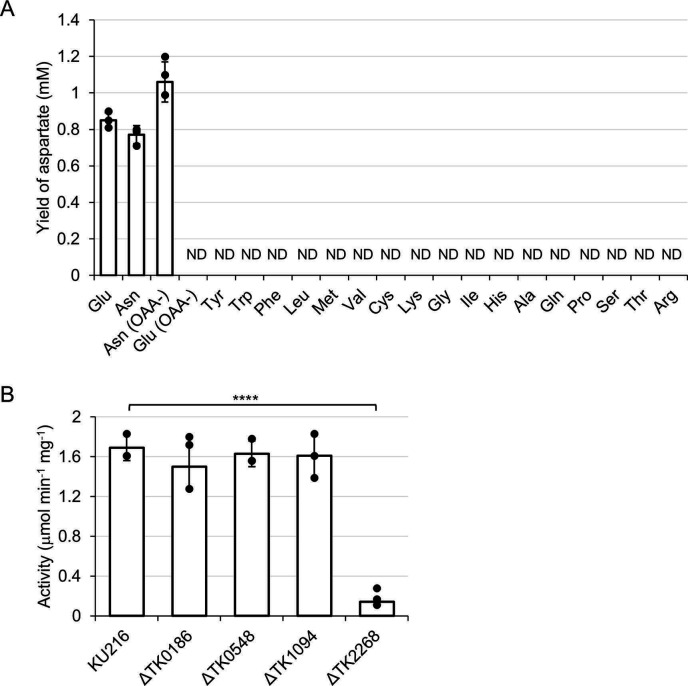


## PORCINE PATHOGENS UNDER REVIEW

Antibiotic resistance has emerged in the porcine and human pathogen *Streptococcus suis*. Lv et al. (e02160-24) review multitarget prevention and treatment options to combat infections.


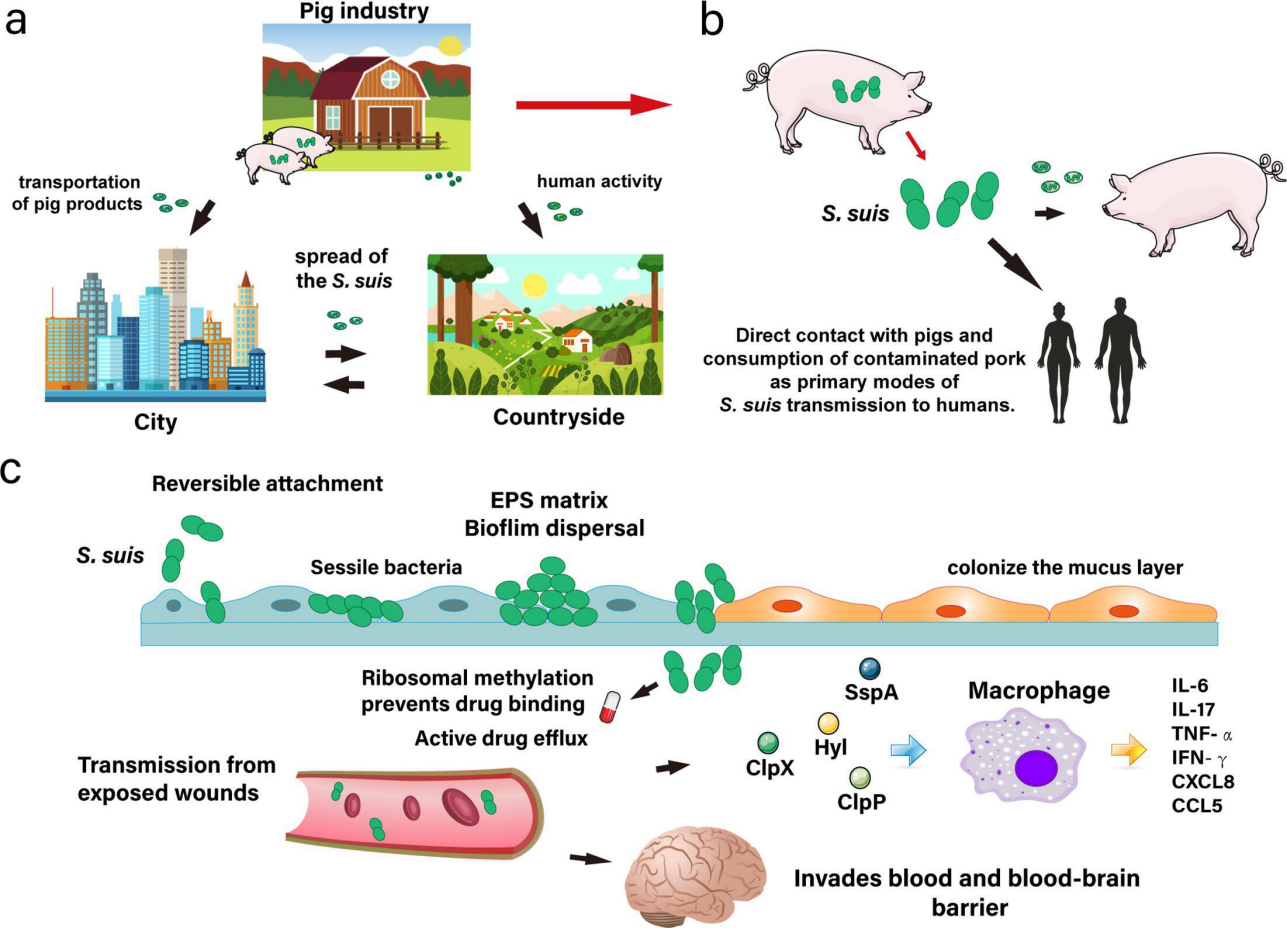


## THE METHANE PROBLEM OF RESTORED WETLANDS

Wetlands are important contributors to carbon sequestration – but they are also the largest natural source of methane. Hamovit et al. (e02161-24) show that wetlands flooded to restore carbon sequestration have a distinct microbial community and a higher potential for emissions.


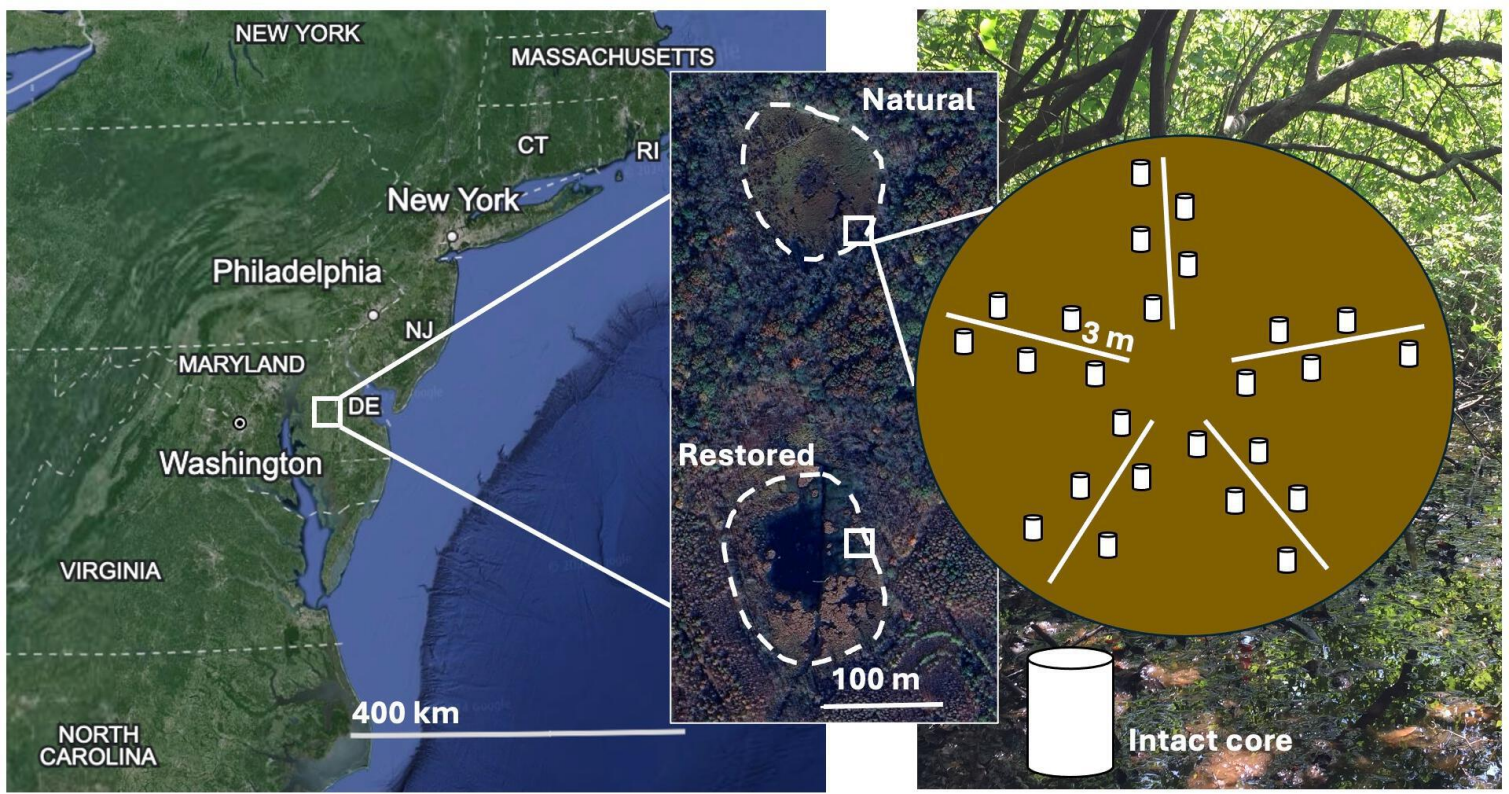


## SALMONELLA SEROTYPING GETS UPGRADED

Deng et al. (e02600-24) share a workflow to predict *Salmonella* serotypes from genome sequencing data. This simpler approach to serotyping provides a much-needed tool for effective foodborne pathogen surveillance and control.